# TAMRA/TAMRA Fluorescence Quenching Systems for the Activity Assay of Alkaline Phosphatase

**DOI:** 10.3390/s17081877

**Published:** 2017-08-15

**Authors:** Akio Shiba, Emiko Kinoshita-Kikuta, Eiji Kinoshita, Tohru Koike

**Affiliations:** Department of Functional Molecular Science, Graduate School of Biomedical & Health Sciences, Hiroshima University, Hiroshima 734-8553, Japan; d165129@hiroshima-u.ac.jp (A.S.); kikuta@hiroshima-u.ac.jp (E.K.-K.)

**Keywords:** alkaline phosphatase, fluorometric analysis, fluorescence quenching, tetramethylrhodamine, *O*-phosphorylethanolamine, pyrophosphate

## Abstract

We introduce two types of fluorescence-quenching assay for alkaline phosphatases (APs) by using a carboxytetramethyl-rhodamine (TAMRA)-labeled phosphate-binding tag molecule (TAMRA-Phos-tag). In the first assay, TAMRA-labeled *O*-phosphorylethanolamine (TAMRA-PEA) was used as an artificial AP-substrate. TAMRA-Phos-tag specifically captured TAMRA-PEA to form a 1:1 complex at pH 7.4; the intensity of the fluorescence peak of the complex at 580 nm (λ_ex_ = 523 nm) was significantly reduced to 32% of the average value for the two individual components as a result of the mutual approach of the TAMRA moieties. As TAMRA-PEA was dephosphorylated by AP, the resulting TAMRA-labeled ethanolamine dissociated and the fluorescence increased in a manner dependent on the AP dose and the time. In the second assay, pyrophosphate (PP), a natural AP-substrate, was used as a bridging ligand to form a dimeric TAMRA-Phos-tag complex. The dimerization reduced the fluorescence intensity to 49% of that in the absence of PP. As pyrophosphate was hydrolyzed to two orthophosphate moieties by AP, the 580-nm fluorescence recovered in a time-dependent manner. By examining the initial slope of this time-dependent fluorescence recovery, we succeeded in evaluating the 50% inhibitory concentrations of orthovanadate toward two AP isozymes under near-physiological conditions.

## 1. Introduction

Alkaline phosphatases (APs, EC 3.1.3.1) are found in a wide range of organisms ranging from bacteria to mammals [1]. These enzymes catalyze hydrolyses of various phosphate compounds and transphosphorylations from phosphorylated species to other molecules [2]. As one of the most commonly assayed enzymes, serum AP is widely used as a clinical indicator for various diseases, including liver dysfunction and cancer [3,4]. Several assays for determining the activity of APs have been reported; these generally involve colorimetric, fluorimetric, chromatographic, radioactive, or electrochemical approaches. Because the optimal pH for the enzyme reaction occurs at alkaline pH values, most in vitro AP assays are conducted at pH values above 8 by using a chromophore-labeled artificial substrate [5,6,7]. However, a few AP assays that use nonlabeled natural substrates, such as nucleotides or inorganic pyrophosphates, at a physiological neutral pH have been reported [8,9,10].

We previously reported that the binuclear metal complex 1,3-bis[bis(pyridin-2-ylmethyl)-amino]propan-2-olato dizinc(II) (Phos-tag) acts as a phosphate-binding tag molecule under near-physiological conditions in aqueous solution at neutral pH [11]. As a result, a number of original analytical methods that use various Phos-tag derivatives have been developed for research on the phosphoproteome [12,13,14,15]. In 2009, the phosphate-capturing ability of an aminocoumarin-functionalized Phos-tag molecule was utilized in the development of a fluorescence resonance energy transfer (FRET) system for assaying the dephosphorylation of a fluorescein-labeled phosphopeptide (a fluorophore-labeled substrate) by bovine intestinal AP [16]. This assay was based on the principle that the aminocoumarin-labeled Phos-tag derivative captures the fluorescent phosphopeptide in preference to its nonphosphorylated counterpart. The formation of a 1:1 complex between the Phos-tag derivative and the phosphopeptide brings the fluorescence donor near the acceptor, resulting in FRET with an efficiency that varies from 47 to 86%, depending on the type of peptide sequence. We applied a similar FRET system to an examination of the reverse reaction: phosphorylation of a fluorescein-labeled peptide substrate by a certain kinase [17]. Moreover, a fluorescence-quenching system that uses a 4-{[4-(dimethylamino)phenyl]-azo}benzoate (dabcyl)-labeled Phos-tag derivative and riboflavin 5′-phosphate (a fluorescent natural substrate) was developed for use in a novel AP-inhibitor screening method [18].

Many fluorescence-quenching systems that use two fluorophores have been developed for the quantitative analysis of biomolecules such as nucleic acids or peptides [19]. Among these quenching systems, a pair of 5-carboxy-*N*,*N*,*N′*,*N′*-tetramethylrhodamine (TAMRA) moieties has been applied in assays of the activities of proteases and reductases [20,21]. If the TAMRA moieties approach one another closely, a ground-state TAMRA/TAMRA complex is formed and the fluorescence of the TAMRA groups is efficiently reduced. The TAMRA fluorophore has the advantages of a pH-insensitive quantum yield, excellent photostability, excitation by visible light, and chemical stability under physiological conditions. In this study, we introduce a novel TAMRA-labeled Phos-tag derivative (TAMRA-Phos-tag), which preferentially captures the AP substrates TAMRA-labeled *O*-phosphorylethanolamine (TAMRA-PEA) or pyrophosphate (PP) at micromolar concentrations. TAMRA-PEA is an artificial substrate for APs, whereas PP is a natural AP-substrate. As a novel fluorimetric analysis of AP activity, we demonstrate a TAMRA/TAMRA quenching system that uses a 1:1 mixture of TAMRA-PEA and TAMRA-Phos-tag under near-physiological conditions. Furthermore, another type of TAMRA/TAMRA quenching system that uses a PP-bridged TAMRA-Phos-tag dimer is described for profiling the inhibition of two types of AP isozyme by the dihydrogen orthovanadate ion (H_2_VO_4_^–^).

## 2. Materials and Methods

### 2.1. Materials

The amino-pendent Phos-tag ligand, *N*-(2-aminoethyl)-6-{[{3-[bis(pyridin-2-ylmethyl)amino]-2-hydroxypropyl}(pyridin-2-ylmethyl)amino]methyl}nicotinamide, was obtained from NARD Chemicals Ltd. (Amagasaki, Japan). 5-Carboxy-*N,N,N′,N′*-tetramethylrhodamine succinimidyl ester (5-TAMRA-NHS) was purchased from ChemPep Inc. (Wellington, FL, USA). 2,2′,2″,2‴-(Ethane-1,2-diyldinitrilo)tetraacetic acid disodium salt (EDTA·2Na), 2-[4-(2-hydroxyethyl)piperazin-1-yl]ethane-1-sulfonic acid (Hepes), and Cosmosil 140C_18_-OPN were purchased from Nacalai Tesque, Inc. (Kyoto, Japan). A basic silica-gel resin, NH-DM 1020, was purchased from Fuji Silysia Chemical, Ltd. (Kasugai, Japan). Crosslinked polystyrene beads (Diaion HP-20) were purchased from Mitsubishi Chemical Corp. (Tokyo, Japan). Bovine intestinal mucosa AP Type VII-S and *Escherichia coli* AP were purchased from Sigma-Aldrich. AP-dose is shown as a conventional enzyme activity-unit as follows: one unit of the phosphatase hydrolyzed 1 µmol of 4-nitrophenyl phosphate per minute at pH 9.8 and 37 °C. TLC was performed on silica-gel plates (No. 5533 or No. 5560; Merck, Darmstadt, Germany). All chemical reagents and solvents were of the highest commercial quality and were used without further purification. All aqueous solutions were prepared by using distilled water.

### 2.2. Apparatus

Visible spectra were recorded on a V-630 spectrophotometer (JASCO Corp., Tokyo, Japan) with a temperature-controlled cell holder at 25.0 ± 0.1 °C. Fluorescence spectra were measured on a FP-8300 fluorescence spectrophotometer (JASCO Corp., Tokyo, Japan) equipped with a Peltier thermostatted cell holder at 25.0 ± 0.1 °C. IR spectra were recorded on a FT/IR-4600 spectrophotometer (JASCO Corp., Tokyo, Japan) at room temperature. ^1^H (500 MHz) and ^13^C (125 MHz) NMR spectra were recorded on an LA500 spectrometer (JEOL Ltd., Tokyo, Japan) at 25 °C: Tetramethylsilane (TMS; δ = 0.0 ppm) in CDCl_3_ containing the TAMRA-labeled Phos-tag ligand, and ^1^H-DMSO (δ = 2.5 ppm) in DMSO-d_6_ containing the TAMRA-labeled *O*-phosphorylethanolamine were used as internal references for the NMR measurements. Measurements of pH were performed with a LAQUA F-72 pH meter with a 9618-10 combination pH electrode (Horiba Ltd., Kyoto, Japan), calibrated by using standard pH buffers (pH 4.01 and 6.86) at 25 °C. High-resolution mass spectroscopy (HRMS) was performed by using an LTQ Orbitrap XL electrospray mass spectrometer (Thermo Fisher Scientific K.K.; Yokohama, Japan) in the positive-ion mode.

### 2.3. Preparation of TAMRA-labeled Derivatives

**Figure 1:**
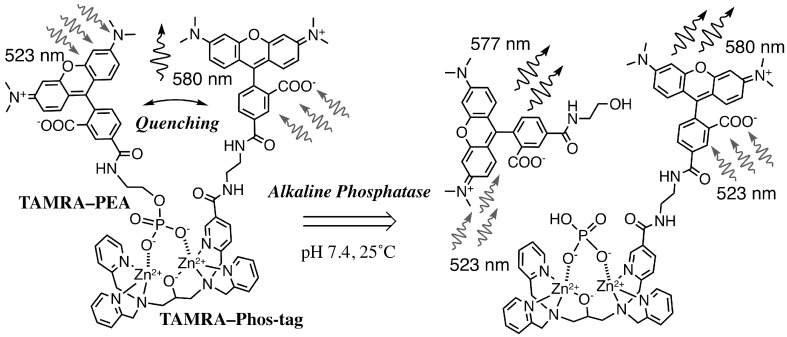
Structure of the TAMRA-Phos-tag complex with TAMRA-PEA and the dephosphorylation of TAMRA-PEA to reduce the fluorescence quenching effect at pH 7.4 in aqueous solution.

TAMRA-labeled Phos-tag ligand: A solution of 5-TAMRA-NHS (96 mg, 0.18 mmol) in CHCl_3_ (5.0 mL) was added to a solution of the amino-pendent Phos-tag ligand (100 mg, 0.18 mmol) in CHCl_3_ (5.0 mL), and the mixture was allowed to react for 12 h at room temperature. The solvent was then evaporated and the residue was purified by column chromatography [basic silica gel, CHCl_3_-MeOH (50:1); resin: NH-DM 1020] to give TAMRA-labeled Phos-tag ligand (Figure 1) as a dark-purple solid; yield: 123 mg (0.13 mmol, 72%). TLC [silica gel plate (No. 5533), CHCl_3_-MeOH (10:1)]: *R*_f_ = 0.50 (one magenta-colored spot). IR (KCl): 3264 (br), 2918, 1750 (COO), 1649 (CON), 1613 (CON), 1594, 1534, 1488, 1433, 1407, 1347, 1305, 1253, 1227, 1188, 1122, 929, 822, 768, 665 cm^–1^. ^1^H-NMR (CDCl_3_): δ = 2.55 (2H, dd, *J* = 8 and 13 Hz, NCCCHN), 2.61 (2H, dd, *J* = 4 and 13 Hz, NCCCHN), 3.03 (12H, s, CH_3_), 3.51 (2H, br s, NCCH_2_N), 3.58 (2H, br s, NCCH_2_N), 3.72–3.85 (8H, m, PyCH_2_), 3.94 (1H, tt, *J* = 4 and 8 Hz, NCCHCN), 6.43 (2H, d, *J* = 3 Hz, ArH), 6.46–6.49 (2H, m, ArH), 6.86 (2H, dd, *J* = 3 and 9 Hz, ArH), 7.05–7.09 (3H, m, PyH), 7.12 (1H, d, *J* = 8 Hz, ArH), 7.21 (1H, d, *J* = 8 Hz, PyH), 7.30 (3H, dd, *J* = 8 and 14 Hz, PyH), 7.50–7.57 (3H, m, PyH), 8.03 (1H, dd, *J* = 2 and 8 Hz, PyH), 8.15 (1H, dd, *J* = 2 and 8 Hz, ArH), 8.43–8.46 (3H, m, PyH), 8.81 (2H, br s, NHCO and ArH), 8.95 (1H, d, *J* = 2 Hz, PyH), 9.22 (1H, br s, NHCO). ^13^C-NMR (CDCl_3_): δ = 40.2, 40.5 (CH_3_), 40.8, 59.0, 59.1, 60.8, 67.4, 97.5, 109.5, 110.8, 122.1, 122.4, 123.2, 126.0, 126.2, 128.3, 130.2, 132.1, 134.0, 135.8, 136.2, 136.5, 146.0, 148.4, 149.0, 154.2, 154.8, 159.3, 159.4, 162.0, 165.8 (CO), 167.5 (CO), 170.2 (CO). HRMS (electrospray, MeOH): *m/z* [M + H]^+^ calcd. for C_55_H_57_N_10_O_6_^+^: 953.4457; found: 953.4442.

TAMRA-labeled *O*-phosphorylethanolamine (TAMRA-PEA): A solution of 5-TAMRA-NHS (96 mg, 0.18 mmol) was added to a pH 8.0 aqueous buffer solution of *O*-phosphorylethanolamine (PEA; 2-aminoethyl dihydrogen phosphate) and NaOH (5.0 mL). The mixture was stirred for two hours at room temperature. 1.0 M aqueous HOAc (2.0 mL) was added and the acidic aqueous solution was passed through a column of Diaion HP-20(Internal diameter: 25 mm, length: 150 mm). The column was then washed with distilled water (300 mL) to remove hydrophilic compounds such as PEA and NaOAc. The remaining TAMRA derivatives, such as 5-TAMRA-carboxylic acid and TAMRA-PEA {2-[6-(dimethylamino)-3-(dimethyliminio)-3*H*-xanthen-9-yl]-5-({[2-(phosphonooxy)ethyl] amino} carbonyl) benzoate} were eluted with MeOH (100 mL). The collected organic phase was concentrated and the residue was purified by C_18_-reverse-phase column chromatography [Cosmosil 140C_18_-OPN, CHCl_3_-MeOH (50:1)] to give TAMRA-PEA as a dark-purple solid; yield: 65 mg (0.12 mmol, 65%). TLC [No. 5560 silica-gel plate; MeOH-H_2_O (1:1)]: *R*_f_ = 0.40 (single magenta-colored spot). IR (KBr): 3419 (br), 2930, 1715 (COO), 1647 (CON), 1603 (CON), 1560, 1535, 1512, 1495, 1410, 1366, 1352, 1191, 1080, 929, 815, 701, 505 cm^–1^. ^1^H-NMR (500 MHz, DMSO-d_6_): δ = 3.02 (12H, s, CH_3_), 3.47–3.52 (2H, br s, CH_2_), 3.68 (2H, br s, HO in a DHO signal), 3.92–3.97 (2H, br s, CH_2_), 6.63 (2H, s, ArH), 6.68 (4H, s, ArH), 7.33 (1H, d, *J* = 8 Hz, ArH), 8.25 (1H, d, *J* = 8 Hz, ArH), 8.48 (1H, s, ArH), 9.25 (1H, br s, NHCO). HRMS (electrospray, MeOH): *m/z* [M + H]^+^ calcd. for C_27_H_29_N_3_O_8_P^+^: 554.1687; found. 554.1688.

### 2.4. Fluorescence Aanalysis

All fluorescence measurements were performed in triplicate or more by using aqueous sample solutions containing 10 mM Hepes-NaOH (pH 7.4) and 0.10 M NaCl. The sample solution (3.0 mL) in a 1-cm-pathlength cuvette (10 × 10 × 45 mm) was continuously stirred with a 3-mm magnetic stirring bar at 25.0 ± 0.1 °C. The slit widths for excitation at 523 nm and emission at 580 nm were both 2.5 nm. In time-course experiments for the AP assay, the excitation was shutter controlled at each data-collection point with a time interval of 1.0 sec. Each AP reaction was monitored for 20 min.

## 3. Results and Discussion

### 3.1. Preparation and Characterization of 5-TAMRA-Labeled Derivatives

**Figure 2:**
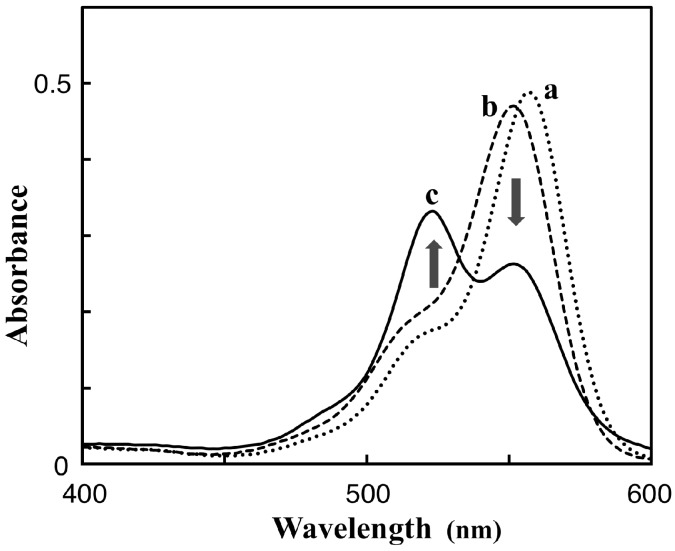
Visible absorption spectra at pH 7.4 (10 mM Hepes-NaOH, 0.10 M NaCl) and 25 °C: (curve **a**) 5.0 µM TAMRA-Phos-tag (dotted curve), λ_max_ = 557 nm (ε = 9.7 × 10^4^ M^−1^·cm^–1^); (curve **b**) 5.0 µM TAMRA-PEA (broken curve), λ_max_ = 551 nm (ε = 9.4 × 10^4^ M^−1^·cm^–1^); (curve **c**) 1:1 mixture of 2.5 µM TAMRA-Phos-tag and 2.5 µM TAMRA-PEA (solid curve), λ_max_ = 523 nm (Abs. = 0.33).

**Figure 3:**
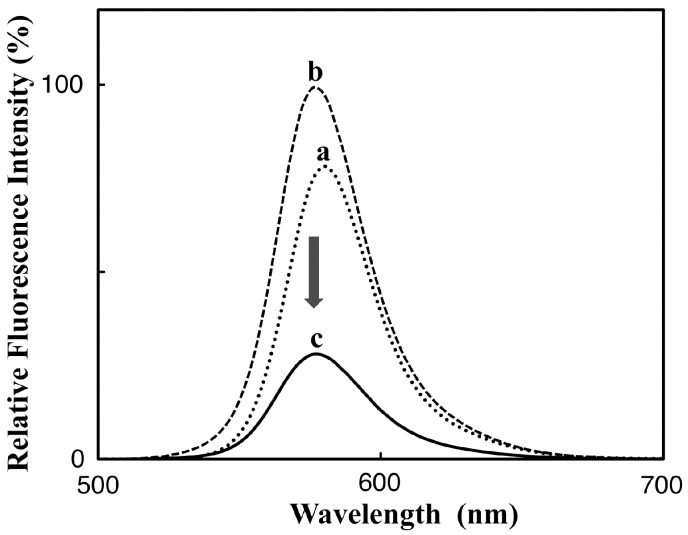
Fluorescence emission spectra (λ_ex_ = 523 nm) at pH 7.4 (10 mM Hepes-NaOH, 0.10 M NaCl) and 25 °C: (curve **a**) 5.0 µM TAMRA-Phos-tag (dotted curve), λ_em_ = 580 nm; (curve **b**) 5.0 µM TAMRA-PEA (broken curve), λ_em_ = 577 nm; (curve **c**) 1:1 mixture of 2.5 µM TAMRA-Phos-tag and 2.5 µM TAMRA-PEA (solid curve), λ_em_ = 577 nm.

The TAMRA-labeled Phos-tag ligand was synthesized by a coupling reaction of an amino-pendent Phos-tag ligand and an amine-reactive NHS derivative of 5-TAMRA in 72% yield (see Materials and Methods, [11]). A stock solution of the dinuclear zinc(II) complex of TAMRA-Phos-tag (60 µM) was prepared by mixing the ligand (60 µM) with 2.5 equivalents of zinc(II) chloride (150 µM) in 10 mM Hepes-NaOH buffer (pH 7.4) containing 0.10 M NaCl at room temperature. In the buffer solution, TAMRA-Phos-tag was stable and showed no change in its absorption spectrum on standing for six months at 4 °C in the dark. By reference to previously reported Phos-tag derivatives [10,17], an analogous structure of the dinuclear zinc(II) complex is show in Figure 1. Aqueous solutions of the complex are bright magenta in color over a wide pH range from 5 to 11. The solubility of TAMRA-Phos-tag is about 80 µM in the buffer solution at room temperature, which is enough to permit spectrophotometric analysis by using a general apparatus with a 1-cm cuvette. The visible absorption spectrum of 5.0 µM TAMRA-Phos-tag in a buffer solution (pH 7.4) containing 10 mM Hepes-NaOH and 0.10 M NaCl at 25 °C is shown in Figure 2 (curve a). The absorption maximum (λ_max_) was observed at 557 nm (ε = 9.7 × 10^4^ M^−1^·cm^−1^) with an absorption shoulder at about 520 nm. The fluorescence spectrum (λ_em_ = 580 nm, λ_ex_ = 523 nm) of the same solution as used to obtain the visible absorption spectrum (Figure 3, curve a) was similar to that of a general amine-bound 5-TAMRA derivative, with an emission peak (λ_em_) at around 580 nm in aqueous solution [22].

TAMRA-labeled phosphorylethanolamine (TAMRA-PEA; Figure 1) was prepared by coupling 5-TAMRA-NHS with an excess of PEA in aqueous buffer solution (pH 8.0) (see Materials and Methods). The spectrophotometric characteristics of TAMRA-PEA were examined in the same buffer system (pH 7.4) as that used for TAMRA-Phos-tag. The visible absorption and fluorescence spectra of 5.0 µM TAMRA-PEA are shown in Figure 2 (curve b) and Figure 3 (curve), respectively. The spectra are similar to those of TAMRA-Phos-tag. An absorption maximum was observed at 551 nm (ε = 9.4 × 10^4^ M^−1^·cm^–1^) with an absorption shoulder at about 520 nm. In the buffer solution; TAMRA-PEA was also stable without change in its absorption spectrum or hydrolysis of the phosphate ester after standing for six months at 4 °C in the dark. The fluorescence spectrum (λ_ex_ = 523 nm) had an emission peak at 577 nm. The greater fluorescence intensity of TAMRA-PEA (Figure 3, curve b) compared with that of TAMRA-Phos-tag (Figure 3, curve a) is associated with the more-intense visible absorption at 523 nm (Figure 2, curve b).

### 3.2. Characteristics of the Phos-Tag-Based Fluorescence-quenching System

We conducted a spectrophotometric analysis of mixed samples containing TAMRA-Phos-tag and TAMRA-PEA in a buffer solution (pH 7.4) containing 10 mM Hepes-NaOH and 0.10 M NaCl at 25 °C. The test solution (3.0 mL) contained TAMRA-Phos-tag and/or TAMRA-PEA at a total concentration of the TAMRA moiety of 5.0 µM. As the molar fraction (MF) of TAMRA-Phos-tag {MF = [TAMRA-Phos-tag]/([TAMRA-Phos-tag] + [TAMRA-PEA])} approached 0.50 from either 0 or 1.0, the visible absorption at 557 nm for TAMRA-Phos-tag (Figure 2, curve a) and at 551 nm for TAMRA-PEA (Figure 2, curve b) decreased, and the intensity of the shoulder absorbance at about 520 nm increased. The largest change in the shape of the spectrum was obtained at MF = 0.50 (TAMRA-Phos-tag and TAMRA-PEA each at 2.5 µM). The visible absorption spectrum of the 1:1 mixed solution is shown in Figure 2, curve c. The absorption peak at 523 nm remained, but with a reduced absorbance of 0.33, and was clearly blue-shifted from those for TAMRA-Phos-tag or TAMRA-PEA alone. The marked changes in the spectrum are consistent with the formation of a 1:1 complex, as shown in Figure 1, in which the TAMRA-TAMRA distance can be estimated to be less than 2 nm by using a general molecular model. Similar changes in visible spectra have been reported for a bis (TAMRA)-peptide and a bis (TAMRA)-disulfide derivative in which the two TAMRA moieties are sufficiently close to one another to form an intramolecular ground-state complex, resulting in efficient static quenching of the fluorescence of TAMRA [20,21]. Consequently, the fluorescence quenching for the TAMRA-Phos-tag system would occur by a similar mechanism as previously reported.

Next, we determined the efficiency of the TAMRA/TAMRA fluorescence quenching in the complexation of TAMRA-Phos-tag with TAMRA-PEA. As is the case of visible absorption, the largest change in fluorescence intensity at 580 nm was observed at MF = 0.50, with an optimized excitation wavelength of 523 nm (see Figure 3). The fluorescence intensity of the 1:1 mixture of TAMRA-Phos-tag and TAMRA-PEA (Figure 3, curve c; each at 2.5 µM) was 32% of the average value of 5.0 µM TAMRA-Phos-tag (Figure 3, curve a) and 5.0 µM TAMRA-PEA (Figure 3, curve b). The 68% decrease in fluorescence intensity is significantly larger than the 45% decrease in the visible absorbance at 554 nm (see Figure 2). As a reference experiment, we removed the zinc(II) ions from the 1:1 complex of TAMRA-Phos-tag and TAMRA-PEA (each at 2.5 µM) by complexation with 1.0 mM EDTA [a strong zinc(II)-chelating agent] at 25 °C. Immediately after the addition of EDTA, the fluorescence with excitation at 523 nm gradually increased and finally reached a steady value after incubation for one hour at 25 °C. The final intensity at 580 nm was almost the same as the average value for 5.0 µM TAMRA-Phos-tag and 5.0 µM TAMRA-PEA, showing that the TAMRA-Phos-tag ligand has no affinity to TAMRA-PEA under the experimental conditions, and therefore phosphate coordination to the zinc(II) ions of the Phos-tag moiety is necessary for fluorescence quenching to occur.

### 3.3. Real-Time Analysis of Dephosphorylation of TAMRA-PEA by Aalkaline Phosphatase

**Figure 4:**
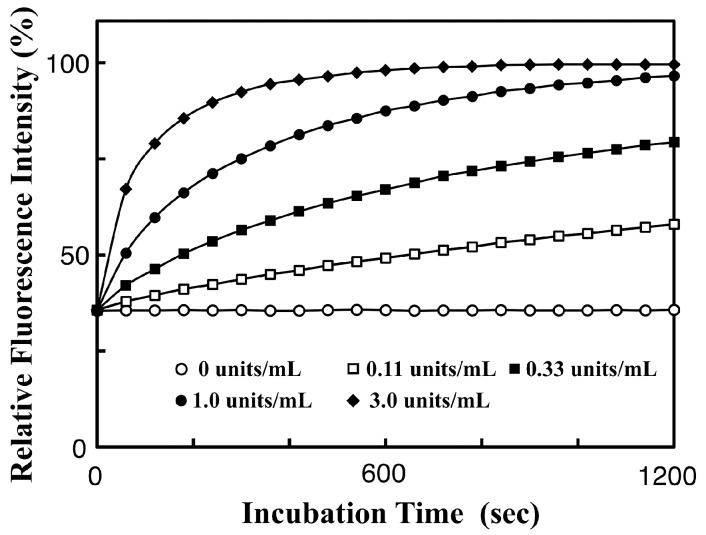
Time course change of the 580-nm fluorescence intensity (λ_ex_ = 523 nm) of an aqueous solution (pH 7.4, 3.0 mL) containing 2.5 µM TAMRA-Phos-tag and 2.5 µM TAMRA-PEA, 0.10 mM MgCl_2_, 0.10 M NaCl, and 10 mM Hepes-NaOH at 25 °C in the absence or presence of bovine intestinal mucosa alkaline phosphatase: 0 units/mL (open circles), 0.11 units/mL (open squares), 0.33 units/mL (closed squares), 1.0 units/mL (closed circles), or 3.0 units/mL (closed diamonds). The typical data are plotted with a time interval of 60 seconds.

We recently reported a FRET-based fluorescence-quenching method for measuring the activity of a bovine intestinal mucosa AP by using a Phos-tag derivative carrying a dabcyl group as a fluorescence quencher and a flavin mononucleotide as a fluorescent AP substrate [18]. In a similar manner to the Phos-tag-based method, the TAMRA/TAMRA fluorescence-quenching system with Phos-tag technology was applied to determine the kinetics of dephosphorylation of TAMRA-PEA by AP (see Figure 1). *O*-Phosphorylethanolamine (PEA) is a natural nonfluorescent substrate of human AP [23]. A real-time analysis of the dephosphorylation of TAMRA-PEA was performed in a stirred 1-cm cuvette at 25.0 ± 0.1 °C. The reaction mixture (3.0 mL) contained 2.5 µM TAMRA-PEA, 2.5 µM TAMRA-Phos-tag, 0.10 mM MgCl_2_, 0.10 M NaCl, and 10 mM Hepes-NaOH (pH 7.4). The reaction was initiated by injection of an appropriate amount of bovine intestinal mucosa AP. The change in sample volume as a result of the injection of the enzyme solution was less than 0.5%. Immediately, the fluorescence intensity at 580 nm (λ_ex_ = 523 nm) increased in a time-dependent manner, and was subsequently measured over 20 minutes. The rate of dephosphorylation increased with increasing concentration of AP. Plots of the fluorescence intensity at one-minute intervals are shown in Figure 4. The dephosphorylation reaction progressed according to almost pseudo-first-order kinetics until the fluorescence intensity reached about half the final plateau value. The enhancement in fluorescence resulting from the elimination of the phosphate group from TAMRA-PEA was 2.9-fold. The initial rates of dephosphorylation of TAMRA-PEA were almost proportional to the amount of AP (0, 0.11, 0.33, 1.0, and 3.0 units/mL). As a reference experiment, we prepared the dephosphorylated TAMRA-PEA (5.0 µM) by incubation with 6.0 units/mL AP for one hour under the same experimental conditions. The resulting solution of TAMRA-labeled ethanolamine showed a similar fluorescence spectrum (λ_em_ = 577 nm) with about 95% of the peak intensity of TAMRA-PEA (5.0 µM). Furthermore, no fluorescence enhancement of a sample solution of TAMRA-PEA (5.0 µM) was observed in the absence of AP under the experimental conditions, even after one hour. More than 95% of AP-activity in the AP assay remained after 1 week from preparation of an AP stock solution stored at 4 °C. The fluorescence change is therefore consistent with the time-dependent dephosphorylation of TAMRA-PEA to produce stoichiometric amounts of inorganic phosphate, which are captured by the TAMRA-Phos-tag molecule (see Figure 1). The aqueous solution of the equimolar mixture of TAMRA-PEA and TAMRA-Phos-tag was also stable without changes in its visible absorption or fluorescence spectra, even after storage for six months in a refrigerator. This TAMRA/TAMRA quenching system can therefore be used as a simple and reliable in vitro assay for AP activity under near-physiological conditions.

### 3.4. Fluorimetric Aanalysis of Pyrophosphate by Using TAMRA-Phos-tag

**Figure 5:**
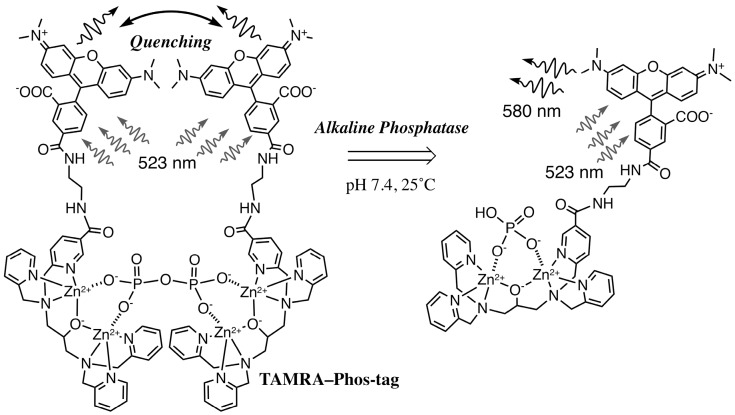
Structure of the dimeric TAMRA-Phos-tag complex linked with pyrophosphate, and the hydrolysis of pyrophosphate to reduce the fluorescence quenching effect at pH 7.4 in aqueous solution.

**Figure 6:**
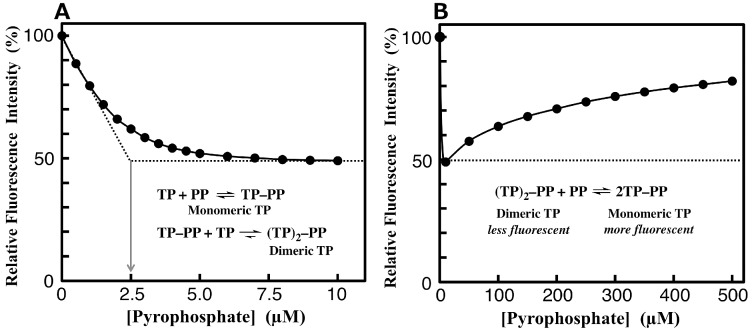
Fluorescence emission response (at 580 nm) of 5.0 µM TAMRA-Phos-tag (TP) to increasing level of pyrophosphate (PP) at pH 7.4 (10 mM Hepes-NaOH, 0.10 M NaCl) and 25 °C. The concentration ranges of pyrophosphate are 0–10 µM for (**A**) and 0–500 µM for (**B**).

We developed an alternate phosphatase assay system that uses only TAMRA-Phos-tag (TP), as illustrated in Figure 5. In this assay, the TAMRA/TAMRA quenching system is based on the formation of a dimeric complex of TAMRA-Phos-tag bridged by a pyrophosphate anion (PP), a natural AP substrate [23]. As with the TAMRA-PEA/TAMRA-Phos-tag system, the intensity of the 557-nm absorbance (ε = 9.7 × 10^4^ M^−1^·cm^−1^) of 5.0 µM TAMRA-Phos-tag decreased with increasing concentration of PP (ε = 7.6 × 10^4^ M^−1^·cm^−1^ at [Pyrophosphate] = 10 µM), pH 7.4 (10 mM Hepes-NaOH and 0.10 M NaCl), and 25 °C (see Appendix). A reference experiment in the presence of 20 µM orthophosphate anion (HPO_4_^2–^) showed no change in the visible absorption. Furthermore, the visible spectrum of 5.0 µM TAMRA-Phos-tag was unchanged in the presence of 1.0 mM EDTA on addition of 10 µM PP under the same conditions. These results indicate that PP acts as a bridging ligand to form a dimeric TAMRA-Phos-tag complex, as shown in Figure 5. In the fluorimetric analysis of the same test solutions, the intensity of the emission at 580 nm (λ_ex_ = 523 nm) significantly decreased in the presence of 10 µM PP (see Appendix). The rate of decrease of 51% for the fluorescence intensity was significantly greater than that of 22% observed for the visible absorbance at 557 nm. The fluorescence intensity with the excitation at 557 nm, which is the absorption maximum wave length of 5.0 µM TAMRA-Phos-tag, was almost twice larger than that with the excitation at 523 nm, but the decrease in the fluorescence intensity at 580 nm (λ_ex_ = 557 nm) in the presence of 10 µM PP was about 25% under the same experimental conditions. A typical relationship for the fluorescence intensity of 5.0 µM TAMRA-Phos-tag and the concentrations of PP (0–10 µM) is plotted in Figure 6A. An intersection value of 2.5 µM for [PP] was obtained from two tangential lines of the decay curve of Figure 6A. The dose-dependent fluorescence quenching showed that the formation of a PP-bridged TAMRA-Phos-tag 1:2 complex [(TP)_2_-PP], and confirmed that the dimer is more stable than the PP-bound monomer (TP-PP) under the experimental conditions (Figure 6A). The greater stability of the dimer indicates that two TAMRA moieties aggregate to form a ground-state complex, as indicated by the change in visible absorption. On the other hand, further addition of a large excess of PP (50–500 µM) caused an increase in the fluorescence intensity (see Figure 6B). On increasing in PP concentration, the intensity of the visible absorption at 557 nm for TAMRA-Phos-tag increased and that of the shoulder absorbance at around 520 nm decreased. The PP-dose-dependent change in the fluorescence intensity and the visible absorption are probably due to partial formation of the 1:1 complex, as shown in the equation in Figure 6B. The gradual increase in fluorescence intensity at higher concentrations of PP provides further evidence that the dimer is more stable than the monomer. Therefore, the PP-dose-dependent fluorescence response at low concentrations of PP can be used in a sensitive AP assay using the natural substrate and TAMRA-Phos-tag (see below).

### 3.5. Phosphatase-Inhibitor Profiling by Using TAMRA-Phos-tag and Pyrophosphate

**Figure 7:**
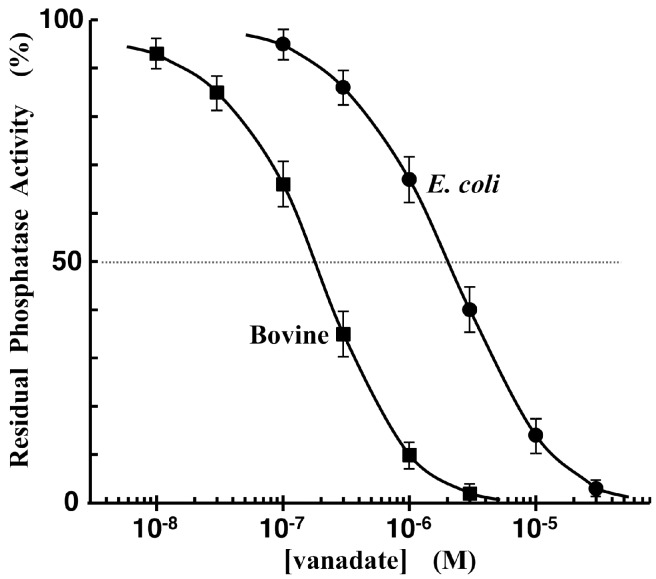
Inhibition curves of the AP reactions in the presence of dihydrogen orthovanadate ion (H_2_VO_4_^–^) at 25 °C. The ordinate represents the residual phosphatase activity ratio (%). The reaction mixture contained 5.0 µM TAMRA-Phos-tag, 2.5 µM pyrophosphate, 0.10 mM MgCl_2_, 0.10 M NaCl, and 10 mM Hepes-NaOH (pH 7.4). The amounts of bovine intestinal AP (closed squares) and E. coli AP (closed circles) were 0.05 units/mL and 0.02 units/mL, respectively.

To further test the possibility of using our Phos-tag-based fluorescence-quenching system as an enzyme assay method, we conducted a real-time analysis of the hydrolysis of pyrophosphate by an AP from bovine intestinal mucosa [24]. As in the case of the TAMRA-PEA/TAMRA-Phos-tag quenching system, the pyrophosphate (PP) hydrolysis reaction can be initiated by the injection of an appropriate amount of the AP. The dose-dependent enzyme activity was examined by an initial-slope method using a test solution (3.0 mL) containing 5.0 µM TAMRA-Phos-tag, 2.5 µM PP, 0.10 mM MgCl_2_, 0.10 M NaCl, and 10 mM Hepes-NaOH (pH 7.4). The time-course changes in the fluorescence intensity at 580 nm (λ_ex_ = 523 nm) were monitored continuously until 10% of pyrophosphate was hydrolyzed. The pseudo-first-order rate constants at 0.025, 0.050, 0.075, and 0.10 units/mL of the AP were evaluated from triple independent experiments to be 3.6 × 10^–4^, 7.1 × 10^–4^, 1.1 × 10^–3^, and 1.4 × 10^–3^ s^–1^, respectively. The rate constants were reproducible within ±10% and were almost proportional to the dose of bovine intestinal mucosa AP. Next, the inhibition of the AP (0.050 units/mL) by the dihydrogen orthovanadate ion (H_2_VO_4_^–^), a competitive inhibitor, was examined under the same experimental conditions. The measurement time for each fluorimetric analysis was less than ten minutes. The residual activity ratios (%) in the presence of the inhibitor were evaluated from the initial rates of the pyrophosphate hydrolysis reaction. The hydrolysis rate decreased with increasing dose of the orthovanadate ion. An inhibition curve was obtained from a plot of the residual phosphatase activity against the concentration of the inhibitor on a logarithmic scale (Figure 7; closed squares). From the resulting sigmoidal inhibition curve, the half maximal inhibitory concentration(*IC*_50_) was evaluated to be 0.18 ± 0.05 µM at pH 7.4 and 25 °C. The *IC*_50_ value of the same AP has been reported to be 0.10 µM by using the dabcyl-Phos-tag quenching system with riboflavin 5′-phosphate [18]. Furthermore, the *IC*_50_ value of E. coli AP was determined to be 2.0 ± 0.5 µM from the same plot (Figure 7; closed circles) by using the TAMRA/TAMRA quenching system [1]. The value for the AP isozyme is about one order of magnitude larger than that of bovine intestinal AP. Therefore, a combination of TAMRA-Phos-tag and inorganic pyrophosphate ion (a natural AP substrate) might be useful in a simple inhibitor profiling of AP isozymes at pH 7.4.

## 4. Conclusions

We have developed a novel application of the Phos-tag-based fluorescence quenching system in the analysis of the activity of AP with an artificial substrate, TAMRA-PEA, or with a natural substrate, pyrophosphate, under near-physiological conditions. For this system, we synthesized a novel fluorescent Phos-tag derivative, consisting of a dizinc(II) complex (Phos-tag) attached to a TAMRA group. The Phos-tag derivative preferentially captured the phosphate group of TAMRA-PEA or pyrophosphate, resulting in efficient fluorescence quenching between the two TAMRA moieties. The TAMRA/TAMRA quenching systems were used in continuous monitoring of the AP reaction. As an application of the pyrophosphate-binding TAMRA-Phos-tag complex, we developed an inhibition assay to assess the *IC*_50_ values of orthovanadate anion for two types of AP isoenzyme. The analysis with pyrophosphate is a relatively simple procedure, involving three aqueous solutions containing a 2:1 mixture of TAMRA-Phos-tag/pyrophosphate, the inhibitor, and the AP, respectively. Real-time analysis of the hydrolysis of pyrophosphate is possible without multiple sampling, and the incubation time for the initial-slope method for AP activity analysis is less than ten minutes per sample. Thus, the principle of this TAMRA/TAMRA fluorescence quenching system can be applied in simple and reliable in vitro profiling of AP to identify inhibitors (or activators) or to analyze their activities; furthermore, it requires only a standard laboratory fluorescence spectrometer.

## Figures and Tables

**Figure A1:**
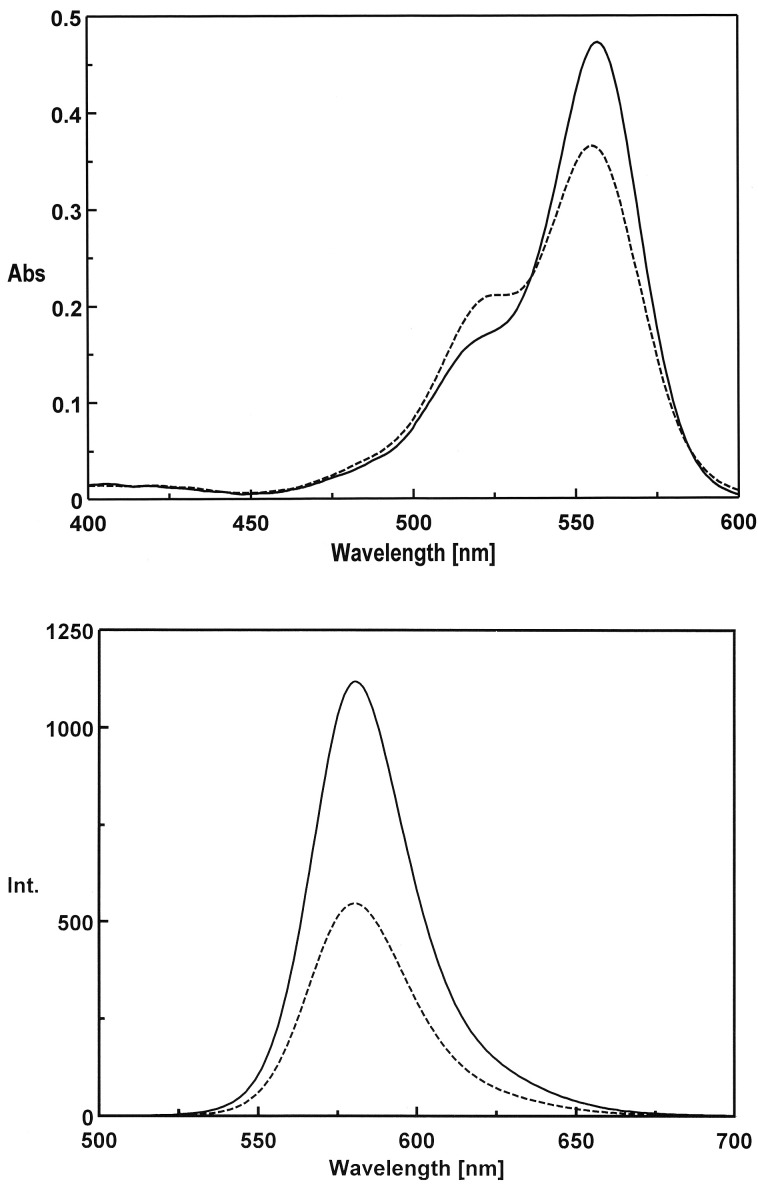
Visible absorption spectra and fluorescence emission spectra for TAMRA-Phos-tag/Pyrophosphate system. (**Top**) Visible absorption spectra at pH 7.4 (10 mM Hepes-NaOH, 0.10 M NaCl) and 25 °C. Solid curve: 5.0 µM TAMRA-Phos-tag, λ_max_ = 557 nm (ε = 9.7 × 10^4^ M^−1^·cm^−1^). Broken curve: 5.0 µM TAMRA-Phos-tag + 10 µM pyrophosphate, λ_max_ = 557 nm (ε = 7.6 × 10^4^ M^−1^·cm^−1^). (**Bottom**) Fluorescence emission spectra (λ_ex_ = 523 nm) at pH 7.4 (10 mM Hepes-NaOH, 0.10 M NaCl) and 25 °C. Solid curve: 5.0 µM TAMRA-Phos-tag, λ_em_ = 580 nm (relative intensity = 100%). Broken curve: 5.0 µM TAMRA-Phos-tag + 10 µM pyrophosphate, λ_em_ = 580 nm (relative intensity = 49%).
